# Application of Forceps

**Published:** 1892-07

**Authors:** 


### Selections and Abstracts.

### APPLICATION OF FORCEPS.

Dr. Henry D. Fry urges, as the only rational method, the application of the forceps to the sides of the head of the child without reference to its position in the pelvis. He refers to a former paper in which it was stated that fifty-one per cent. of prominent obstetricians followed this rule, while thirty-five per cent. applied the blades in transverse diameter of the mother's pelvis without reference to the position of the head, and eleven per cent. observed no rule and followed either method. He admits that had the great body of the profession been consulted, the majority would be found to apply the forceps according to the German method, and also that in some cases it may be and is impossible to do otherwise. Certainly the difficulties of application are increased when the first method is chosen, and it would be better for a beginner to resort to the second until some facility is acquired.

In France it is the practice to apply the forceps to the sides of the head even when transverse at the brim, and the ideal method of extraction is to apply the instruments in such a manner that during traction the fœtal head is free to execute all the movements that would occur were the labor normal. To accomplish this it is necessary:

1. To grasp the sides of the head with the blades.
2. To make traction in the axis of the pelvic canal.
3. To secure mobility of the head during its passage, by the use of Tarnier forceps. The Hodge style of forceps should not be used when their application is made without reference to the child's head, and the Simpson style (Elliot) should not be used when their application is to be made to the sides of the head.

Dr. Fry's conclusions are:

1. Anæsthetize the patient and place her in proper position—buttocks well over the edge of the bed, and each limb supported by an assistant.

2. Ascertain the position of the head, introducing within the vagina two or three fingers, or if necessary, the whole hand.

3. Apply the blades of a Hodge type of forceps to the sides of the head, with the concave edge directed toward the occiput If for any reason this can not be accomplished, withdraw the instrument and substitute a Simpson (or Elliot) passing the blades to the side of the pelvis. While making traction with this method, watch for anterior rotation of the occiput, and encourage it in some cases by re-applying the blades to better advantage.

4. Make every effort to secure antiseptic condition during the operation. The fingers, hands and forearms of the operator, the external genitalia and vagina of the patient, the instruments and the hands of the assistants, should be clean and aseptic.—*Am. Jour. Obst.*

